# A double-blinded randomised controlled trial of vitamin A drops to treat post-viral olfactory loss: study protocol for a proof-of-concept study for vitamin A nasal drops in post-viral olfactory loss (APOLLO)

**DOI:** 10.1186/s40814-023-01402-2

**Published:** 2023-10-12

**Authors:** Kala Kumaresan, Sara Bengtsson, Saber Sami, Allan Clark, Thomas Hummel, James Boardman, Juliet High, Rashed Sobhan, Carl Philpott

**Affiliations:** 1 Norwich Medical School, University of East Anglia, Norwich, UK; 2 Norfolk & Waveney ENT Service, James Paget University Hospital NHS Foundation Trust, Great Yarmouth, UK; 3 School of Psychology, University of East Anglia, Norwich, UK; 4 Technical University, Dresden, Germany; 5 Fifth Sense, Bicester, UK; 6 Norwich Clinical Trials Unit, University of East Anglia, Norwich, UK

**Keywords:** Smell, Vitamin A, Olfactory dysfunction, Randomised controlled trial

## Abstract

**Background:**

Smell loss is a common problem with an estimated 5% of the population having no functioning sense of smell. Viral causes of smell loss are the second most common cause and the coronavirus (COVID-19) pandemic is estimated to have caused 20,000 more people this year to have a lasting loss of smell. Isolation, depression, anxiety, and risk of danger from hazards such as toxic gas and spoiled food are all negative impacts. It also affects appetite with weight loss/gain in two-thirds of those affected. Phantosmia or smell distortion can also occur making most foods seem unpalatable. Smell training has been tried with good results in the immediate post-viral phase. Evidence behind treatment with steroids has not shown to have proven effectiveness. With this, a key problem for patients and their clinicians is the lack of proven effective therapeutic treatment options. Based on previous studies, there is some evidence supporting the regenerative potential of retinoic acid, the metabolically active form of vitamin A in the regeneration of olfactory receptor neurons. It is based on this concept that we have chosen vitamin A as our study comparator.

**Aim:**

To undertake a two-arm randomised trial of intranasally delivered vitamin A vs no intervention to determine proof of concept.

**Methods/design:**

The study will compare 10,000 IU once daily Vitamin A self-administered intranasal drops versus peanut oil drops (placebo) delivered over 12 weeks in patients with post-viral olfactory loss. Potentially eligible patients will be recruited from the Smell & Taste Clinic and via the charity Fifth Sense. They will be invited to attend the Brain Imaging Centre at the University of East Anglia on two occasions, 3 months apart. If they meet the eligibility criteria, they will be consented to enter the study and randomised to receive vitamin A drops or no treatment in a 2:1 ratio. MRI scanning will enable volumetric measurement of the OB and ROS; fMRI will then be conducted using an olfactometer to deliver pulsed odours—phenethylalcohol (rose-like) and hydrogen sulphide (rotten eggs). Participants will also perform a standard smell test at both visits as well as complete a quality-of-life questionnaire. Change in OB volume will be the primary outcome measure.

**Discussion:**

We expect the outputs of this study to enable a subsequent randomised controlled trial of Vitamin A versus placebo. With PPI input we will make the outputs publicly available using journals, conferences, and social media via Fifth Sense. We have already prepared a draft RCT proposal in partnership with the Norwich Clinical Trials Unit and plan to develop this further in light of the findings.

**Trial registration:**

ISRCTN registry 39523. Date of registration in the primary registry: 23rd February 2021.

## Introduction

### Background and rationale

#### The epidemiology and impact of olfactory disorders

Loss of smell is a common complaint in adults and yet its impact has been underestimated. Anosmia, complete loss of smell, is thought to affect at least 1% of the global population with the overall estimated prevalence of olfactory disorders being between 1 and 20% [1–4]. Based on European estimates, anosmia is more prevalent than profound hearing loss or blindness in the UK [5]. Recent evidence from several population studies shows that anosmia rather than hearing or sight loss, is an independent risk factor for a shortened life span [6–10], with this sensory loss acting as a marker of cumulative toxic environmental exposures [11]. Causes for olfactory loss are varied but the main diagnostic groups include sinonasal disease (62%) and post-viral olfactory loss (PVOL) (11%) [12, 13]. There is now an emerging new cluster of patients with PVOL—those infected with the SARS-CoV-2 virus as part of the global pandemic. With smell loss now an official World Health Organisation symptom of COVID-19, evidence has clearly shown that over 60% of those contracting the virus are affected by this symptom [14, 15]. This means that over 1 billion people have experienced smell loss due to COVID-19 globally to date. The majority of those affected appear to recover their sense of smell within 4 weeks of the onset, but current data suggests that 10–17% have continued smell loss and do not recover spontaneously [16, 17]; based on the UK infection rate (20th April 2021), this means an estimated 250,000 people now have PVOL due to COVID-19.

Due to a lack of high-quality therapeutic trials in this area, there is wide variation in clinical practice and little consistent information provided by clinicians to patients on prognosis or treatment, leaving clinicians uninterested in olfactory disorders and patients without further treatment options [18]. Patients can now be encouraged to adopt the rehabilitation technique known as smell training [19]; however, despite this, patients remain in need of effective additional therapeutic options [20]. Patients find the motivation to undertake smell training is harder if no residual olfactory function is left or if they suffer from parosmia, whereby they experience unpleasant sensations when in contact with the stimulus.

PVOL is diagnosed when patients report smell loss that starts with an upper respiratory tract infection and does not recover when the other symptoms disappear; in addition to the presenting timeline, patients lack objective examination findings such as those found in chronic rhinosinusitis (CRS) but show reduced or absent smell performance on psychophysical smell testing. Our previous work using a patient survey (*n* = 496) and a qualitative study showed the major impact on quality of life in people with olfactory loss of all causes in the UK [21, 22]. In specific response to their olfactory impairment, they reported high rates of depression (49%), anxiety (47%), impairment of eating experience (95%), isolation (64%), and relationship difficulties (59%). These findings have been replicated in other studies [23, 24] and patients feel that they have not been managed well and that their condition is usually trivialised [22, 25]. Most patients suffer a loss of flavour perception which can adversely affect their appetite, but this can be made even worse if distortions in their sense of smell (such as parosmia) co-exist (67% of PVOL sufferers) [26, 27]. They often adopt poor dietary habits with negative impacts on their nutritional status and global health, as they tend to eat a less varied diet [27–29]. Many sufferers are also concerned about domestic hazards: 72% of the subjects in an online survey of 1000 affected individuals [30] were concerned about the inability to detect a gas leak or a fire and this resulted in dangerous situations for some subjects [31]. Other subjects with reduced olfactory acuity also experienced adverse effects due to exposure to undetected volatile chemicals [30].

#### Current standard of care for PVOL

During a typical episode of PVOL, health service contacts for such a patient will comprise several GP consultations, between one and three outpatient appointments, and possibly a prescription of corticosteroids. Assuming the rate of depression in these patients is 60% (see above) and even if only 10% of these people receive treatment with antidepressants, then we estimate the annual cost could be close to £10 million. Our public and patient involvement group discovered that the UK Benefits Agency would consider this problem significant in relation to an application for a disability living allowance. Our prior work has also demonstrated difficulties smell loss patients have in accessing healthcare [32].

Loss of smell was considered in the *Generate* research prioritisation agenda in 2015 [33] but the priority was given to CRS as the leading cause of olfactory dysfunction. Guidelines for sinonasal disease exist [34] and research into improving treatment pathways is underway (National Institute of Health Research (NIHR) funded MACRO Programme, Philpott CI) [35]. As outlined above, other causes of smell loss that are not caused by a blockage in the nose, such as PVOL, need more treatments developed to help clinicians provide evidence-based treatments to patients [13]. Although there have been studies exploring the use of various formulations, routes, and doses of steroids, no large randomised controlled trials focused on this subset of patients have been conducted. In a randomised controlled trial by Seo et al. [36], 40 mg of oral prednisolone as monotherapy or combination with 80 mg of ginkgo biloba for 4 weeks was shown to have significant improvement in olfactory function. However, this study did not include a control placebo group to ascertain if the improvement was statistically significant in comparison to an untreated group. A recent survey of 120 patients with PVOL showed that 48% had received no treatment whatsoever, with 35% receiving nasal steroids and 18% oral steroids; none reported the effectiveness of these steroid-based interventions [37]. The recent position paper on olfactory dysfunction concluded [13]: “When considering use of systemic corticosteroids, the risk of side effects must be taken into account. At present, evidence-based guidelines regarding the acceptable frequency of systemic corticosteroid use do not exist. It therefore falls to the individual clinician to exercise the appropriate prudence, particularly in cases of non-CRS related olfactory loss, where the evidence supporting steroid use is poor.” Recently a consensus document by the Clinical Olfactory Working Group concluded that oral steroids should be avoided other than to exclude sinonasal inflammatory disease and some favoured using Vitamin A drops [20].

#### Pathophysiology of olfactory dysfunction and rationale for objective assessments

The location of olfactory receptor neurons (ORNs) is unique, in that they are more likely to suffer damage than the protected nerves of the other senses, being exposed to the external environment and subjected to various external agents including pathogens, pollutants, and dust. The consequent damage to the olfactory neuroepithelium can lead to neurodegeneration [38] and related reduction in olfactory bulb volume (OBV) [39, 40] and right orbital sulcus volume [41]. These changes imply reduced neural activation in the amygdala/temporal piriform and insular cortices [42], brain areas well known to be active during passive perception of odours [43, 44]. Although the olfactory system has a unique capacity to regenerate [45], this process can fail, as occurs following viral injury to the ORNs.

It is the group of viruses that give rise to the common cold and other respiratory tract infections which are thought to be the pathological agent in causing the olfactory loss. This includes rhinoviruses (30–50%), parainfluenza (5%), Coronavirus (10–15%), Influenza (5–15%), Coxsackie (< 5%), adenoviruses (< 5%), and respiratory syncytial viruses (10%); however, there are over 200 viruses in total that produce upper respiratory tract infections [46]. The frequency of viruses found in the studies that have examined the aetiological agents vary with parainfluenza virus common in some (e.g. 88%) [47–49], with others showing differing results and giving prominence to Epstein-Barr virus, rhinovirus, and coronavirus [50].

The pathophysiological mechanism usually involves the virus hijacking the cellular apparatus, but the exact details may depend on the actual virus implicated. Rhinovirus, for instance, causes selective neutrophil and monocyte recruitment to occur. The inflammatory cascade that ensues includes an increase in bradykinin, cytokine, chemokine, and sICAM-1 concentrations [51]. The response in an immunocompetent individual involves T-lymphocyte activation allowing the viral pathogen to be eliminated.

With specific respect to the olfactory apparatus, these viruses appear to cause some partial loss of receptors in the olfactory epithelium. Ultrastructurally, studies have revealed a decrease in the number of olfactory receptor cells and nerve bundles with squamous metaplasia occurring in a few cases [52]. This reduction in the number of ciliated olfactory receptors means at the epithelial surface there is a lack of dendrites and vesicles, therefore, a decrease in the area available for odour molecule detection [53].

Changes in OBV have been shown to correlate with changes in smell performance on psychophysical testing [54], thus we have chosen this as a primary outcome measure for this proof-of-concept study. Significant relationships have also been shown between the volume of key olfactory areas and psychophysical testing including the piriform cortex, orbitofrontal cortex (OFC), and the insular cortex [55]. Prior work by Suebert et al. showed a strong connection between right orbitofrontal cortex volume and general olfactory performance [41]. This demonstrated that larger grey matter volume in the orbital sulcus was the key variable that could be measured in this respect. Therefore, in summary, we have proposed OBV as the primary outcome measure and right orbital sulcus volume as a secondary measure.

#### Explanation for choice of comparators

Vitamin A offers a potential treatment option for olfactory loss due to ORN damage. It is metabolised to retinoic acid (RAc), which as a transcription regulator, is important in tissue development and regeneration [56]. Embryogenesis and ORN regeneration appear to include RAc signalling [57]. When Vitamin A is converted to retinoic acid, it is thought to control olfactory progenitor cell differentiation; therefore it will prevent exhaustion of the stem cell supply or accumulation of non-functional immature neurons [58]. This regeneration of mature neurons from stem cells in adults is limited to the olfactory neuroepithelium, which produces new ORN, and to the subgranular zone, where new granule cells are supplied to the dentate gyrus of the hippocampus in the brain; interneuron production for the olfactory bulbs are also possibly influenced by these stem cells causing an effect in the subventricular zone of the brain [59]. Therefore, it is theorised that topical vitamin A treatment will encourage regeneration of the olfactory epithelium which is damaged by respiratory viruses responsible for the common cold and help to restore the sense of smell in sufferers.

A recent systematic review we conducted identified 4 previous studies that included vitamin A as a treatment modality for olfactory loss [60]. The first, a case series of 56 patients, described some subjective responses to high-dose systemic therapy given by injection, but this was a mixed group of patients with differing aetiologies [61]. A subsequent study for 37 patients with liver cirrhosis treated with *oral* vitamin A demonstrated 2–fivefold improvement in performance on threshold tests for 4 odours as a by-product of the vitamin A treatment [62]. More recently, a double-blind placebo-controlled trial using *oral* vitamin A at the same dose as the previous study of 10,000 IU per day for 3 months was carried out in 52 patients with PVOL and post-traumatic smell loss. However, no obvious treatment effect for oral delivery was shown [63].

In 2017, a pseudo-randomised clinical trial of vitamin A conducted in Germany, this time delivered intranasally at a dose of 10,000 IU per day for 8 weeks, showed that in 124 patients with PVOL, a minimum clinically important difference in olfactory function was seen in 37% of those receiving vitamin A compared to 23% receiving smell training alone [64]. Key attributes of this study included beneficial response in patients with PVOL, apparent evidence of patient safety, and acceptability at the stated dose, duration, and delivery mechanism (intranasally) with minimal side effects evident. Due to unbalanced treatment groups and the pseudo-randomisation, the study lacked scientific rigour our funder (National Institute of Health Research) requested further proof of concept evidence for intranasal Vitamin A.

The dosing regimen proposed below has been based on the previous study undertaken by Hummel et al. [64] and should reduce the potential for adverse effects. Our systematic review allowed for reflection on other options to be considered in this study: corticosteroids (see above), oral theophylline, oral alpha-lipoic acid, and intranasal sodium citrate [60]. The key advantage of taking vitamin A intranasally is the avoidance of oral administration and potential systemic side effects. The alternative use of theophylline requires invasive and more costly blood monitoring with potentially more unpleasant side effects. The effect of sodium citrate in our recent trial was transient with 1 in 3 responding in the active treatment arm but with a need for more data on long-term benefits [65]. The potential benefits of alpha-lipoic acid were based on only 14 patients improving in one small trial [66]. Peanut oil is the base for the vitamin A drops and will therefore act as the placebo.

#### Rationale for this study

This study aims to provide a scientific rationale that will facilitate a larger randomised controlled trial (RCT). Pending the outcome of this study, we would anticipate moving straight to an RCT proposal as soon as is practically possible following the data analysis. The underlying premise is to provide a low-cost but effective treatment option for patients with PVOL in the future which in the wake of the COVID-19 pandemic is needed now more than ever.

## Objectives

### Primary objective

To undertake a two-arm randomised controlled trial of intranasally delivered vitamin A versus placebo (peanut oil) to determine proof of concept.

### Secondary objectives

1. To assess the impact of vitamin A on the olfactory bulb and right orbital sulcus volume using MRI volumetric data and white matter structural connectivity between these brain areas with diffusion MRI (dMRI).
2. To assess the impact of vitamin A on neural activation in the amygdala, temporal piriform, and insular cortices as indicated by an average signal increase of 0.9 in the primary olfactory cortex [67] using fMRI
3. To determine the impact of intranasally delivered vitamin A on psychophysical smell test scores
4. To assess the impact of intranasally delivered vitamin A on quality of life.

## Trial design

### Overall design

The study will be conducted as a two-arm randomised placebo-controlled trial comparing 10,000 IU once daily Vitamin A self-administered intranasal drops versus peanut oil drops delivered over 12 weeks in patients with post-viral olfactory loss.

The UK Medicines and Health Regulation Authority has confirmed study proposed is a mechanistic study and not classified as a CTIMP and therefore does not fall under the need for Clinical Trials Authorisation.

## Methods

### Study setting

The study will take place at the University of East Anglia Brain Imaging Centre (UBIC) with recruitment of patients from the tertiary referral Norfolk Smell & Taste Clinic (TNSTC) located at the James Paget University Hospital (JPUH).

### Eligibility criteria

Adult patients presenting to ENT surgeons with symptoms of anosmia/hyposmia with or without parosmia with no conductive cause identified for their symptoms and a clear history of a preceding upper respiratory tract infection (including COVID-19) that has resolved clinically leaving the olfactory disorder.

The eligibility criteria for this trial have been carefully considered and are the standards used to ensure that only medically appropriate participants are entered. Participants not meeting the criteria should not be entered into the trial for their safety and to ensure that the trial results can be appropriately used to make future treatment decisions for other people with similar diseases or conditions. It is therefore vital that exceptions are not made to these eligibility criteria.

Participants will be considered eligible for enrolment in this trial if they fulfil all the inclusion criteria and none of the exclusion criteria as defined below.

### Trial participant inclusion criteria

A partial or total loss of smell due to post-viral olfactory loss as confirmed on history, examination, and with a smell test (TDI) score of < 31/48 and within 3 years [68] of the precipitating viral infection.

Participants must have received at least 2 doses of any UK government-recognised COVID-19 vaccination, at least 2 weeks before baseline visit.

### Trial participant exclusion criteria

1. Participants with a history of
  a Chronic rhinosinusitis with/without nasal polyposis
  b Severe nasal septal deviation
  c Major prior head injury
  d Congenital olfactory loss
  e Use of concurrent intranasal medications or possible medications known to affect olfaction
  f Chronic renal disease
  g Chronic hepatic disease
  h Allergy to peanuts, soy, or vitamin A (drops contain peanut oil)
2. Significant medical, surgical, or psychiatric disease that in the opinion of the PI would affect subject safety or influence the study outcomes
3. Currently taking oral vitamin A supplements, anticoagulants, or tetracyclines
4. Age of less than 18 or over 65
5. Pregnant women and women of childbearing age not using an effective contraceptive
6. Participants unsuitable for MRI due to metal implants, such as pacemakers, etc., as is standard for MRI scanning or who move excessively during scanning.
7. Evidence from endoscopy or the initial MRI scan of:
  a Participants with any endoscopic findings of:
    i Chronic rhinosinusitis with/without nasal polyposis
    ii Severe nasal septal deviation (preventing passage of 4 mm endoscope)
    iii Other inflammatory sinonasal disease
  b Participants with MRI changes indicating oedema in the sinuses and/or olfactory clefts
8. Any participant with a combined OBV of > 85 mm^3^ will be excluded as it is unlikely they will demonstrate a significant increase in overall volume based on previous studies of OBV [69].
9. Participation in another trial in the last 4 months.

### Co-enrolment guidance

Concurrent participation in clinical trials of investigational medical products is not permitted. However, participants may be entered into other observational studies given prior agreement from the CI of both studies. Pre-trial routine assessments to assess eligibility will be undertaken.

### Screening procedures and pre-randomisation investigations

Written informed consent to enter and be randomised into the trial is obtained from participants, after an explanation of the aims, methods, benefits, and potential hazards of the trial and before any trial-specific procedures. The only procedures performed in advance of written informed consent being obtained are those that would be performed on all patients in the same situation as a usual standard of care.

Patients will have attended TNSTC and undergone a nasal endoscopy and the Sniffin’ Sticks olfactory test (to determine their TDI score) as standard of care. They will then attend their baseline visit at the UBIC. See recruitment details below; these arrangements may depend on the influence of the COVID-19 pandemic. If necessary, in order to avoid the usual endoscopic examination, potential participants may be screened by telephone and if they do not have other rhinological symptoms (such as nasal blockage, rhinorrhoea, and facial pressure), then they will proceed into the study, with the caveat that if the subsequent MRI scan shows inflammatory sinonasal disease, they will be excluded from the study at that point and replaced with another recruit in order to achieve the planned sample size. We anticipate the chances of finding sinonasal disease after screening will be 3% or less [70], and thus may only affect 1 or 2 participants. Women of childbearing age will be asked to confirm that they have a suitable means of contraception and present a negative pregnancy test result prior to being permitted to enter the trial.

### Interventions

Study participants will take vitamin A drops (Vitadral Aristo Pharma GmbH, Berlin, Germany) self-administered intranasally via a dropper at a dose of 10,000 IU (2 drops per nostril) once daily for 12 weeks. The dose and duration are based on the recent study by Hummel et al. [64]. All participants will receive the drops via the UEA APOLLO Research Associate once eligibility is confirmed. The participants will be taught by the research associate how to apply the drops using the Kaiteki position and a leaflet and a video will also be available for them to look at in their own time via the research team’s website (https://rhinology-group.uea.ac.uk/rhinology-and-ent-research-group). The Kaiteki position is a way of ensuring the drops roll into the olfactory cleft at the roof of the nose where the smell receptors are located [71]. Both vitamin A and peanut oil drops for the placebo arm will be received and administered in the same way.

### Arm A

Vitadral Topfen Oral Drops (Aristo Pharma) Vitadral Aristo Pharma GmbH, Berlin, Germany, to be taken daily for 12 weeks, self-administered intranasally via a dropper at a dose of 10,000 IU once daily (2 drops per nostril).

Dispensed by the research Associate after the trial participant is screened and eligibility is confirmed.

The threshold for adherence will be 80% allowing for minor interruptions (of no more than 7 days) of treatment. Participants will also be expected to take the total daily dose of 10,000 IU on at least 80% of occasions.

### Arm B

#### Matched placebo (peanut oil)

Daily application for 12 weeks. Self-administered intranasally via a dropper at a dose of 2 drops to each nostril once daily.

Dispensed by the research associate after the trial participant is screened and eligibility is confirmed.

Equivalent adherence rules will apply to both arms.

All participants will also continue to receive treatment as usual in addition to trial interventions.

### Accountability

Criteria for discontinuing or modifying allocated interventions for a given trial participant:

- Patient experiencing uncomfortable side-effects
- Patient develops a contraindication to MRI scanning, e.g. has a pacemaker fitted

### Compliance and adherence

Regular contact with participants by the research team will seek to ensure that they remain engaged with the study. A trial website will also provide support videos and feedback for nasal drop use. Email reminders every two weeks and follow-up phone calls to participants will aim to ensure that a minimum of 80% of the 12-week course of drops is completed and obtain feedback on any local side-effects.

### Contraindications

- A combination of vitamin A and retinoic acid or its chemical derivatives (e.g. medicinal products for the treatment of skin diseases) should be avoided, as there is a risk of vitamin A overdosing.
- With simultaneous use of high doses of vitamin A and drugs to inhibit blood coagulation (dicumarol, warfarin), the anticoagulant effect can be increased.
- The simultaneous use of antibiotics such as tetracyclines and vitamin A can lead to an increase in intracranial pressure.
- Neomycin (an antibiotic) and cholestyramine and colestipol (medicines used to lower blood lipids) can inhibit vitamin A absorption in the intestine.
- Due to the already established benefit of olfactory training (OT) in PVOL [72], study participants will be asked to refrain from undertaking OT during the study period. Other intranasal treatments will also be discouraged during the study period.

### Overdose of trial medication

Hypervitaminosis is unlikely due to the topical delivery of treatment and was not evident in participants of the previous study. Therapeutic measures in the event of an overdose would be to discontinue the preparation and contact the study team.

### Protocol treatment discontinuation

In consenting to the trial, participants are consenting to trial treatments, trial follow-up, and data collection. However, an individual participant may stop treatment early or be stopped early for any of the following reasons:

- Unacceptable treatment toxicity or adverse event
- Inter-current illness that prevents further treatment
- Any change in the participant’s condition that in the clinician’s opinion justifies the discontinuation of treatment
- Withdrawal of consent for treatment by the participant

As participation in the trial is entirely voluntary, the participant may choose to discontinue trial treatment at any time without penalty or loss of benefits to which they would otherwise be entitled. Although not obliged to give a reason for discontinuing their trial treatment, a reasonable effort should be made to establish this reason, whilst remaining fully respectful of the participant’s rights.

Participants who discontinue protocol treatment, for any of the above reasons, should remain in the trial for the purpose of follow-up and data analysis.

### Outcomes

**Table:** 

Primary	Primary outcome measure: Olfactory bulb volume (OBV) (on MRI scan)
Secondary	• Right orbital sulcus volume (MRI scan) • Blood-oxygen-level-dependant (BOLD)-signal in primary olfactory areas; amygdala, piriform, and insula (fMRI scan) • Cerebral blood flow (CBF) in primary olfactory areas; amygdala, piriform, and insula • Psychophysical smell test (TDI) score • Olfactory disorders questionnaire (ODQ) score • Brain-derived neurotrophic factor (BDNF) (pg/ml)

### MRI scan OBV and sulcus measurements and FMRI changes

For participants in both arms of the study, MRI readings will be taken at baseline and at 12 weeks to allow for olfactory bulb measurement and the use of dynamic changes on fMRI. Each visit will be conducted in the UEA Brain Imaging Centre (UBIC) at the same time of day each time to avoid issues of circadian rhythm. Individual T1-weighted MRI images will be acquired for volumetric analyses of grey matter changes. T2-weighted BOLD imaging sequences will be acquired for analyses of functional changes in the resting state and while participants passively perceive odours (phenethyl alcohol (rose-like pleasant smell) and hydrogen sulphide (rotten eggs -unpleasant) and odourless air in repeated trials. Each stimulus will be delivered through an olfactometer for 3 s per trial, followed by a variable delay of 4–10 s after which the participant will perform a three-alternative forced-choice response indicating which odour they perceived on an MRI-compatible button box. Participants should expect to take 1.5 h for the fMRI. Additionally, diffusion tensor imaging (DTI) sequences will be deployed to investigate white matter fibre integrity and 3-min-long ASL sequence will be executed to measure cerebral blood flow. The olfactometer enables a supply of warmed odourised air flows with a delivery hose to the nose that contains no ferro-magnetic materials, thus compatible with an MRI scanner (Burghart, Wedel, Germany).

### Psychophysical smell test score and ODQ

All participants will undergo the Sniffin’ Sticks smell test at baseline (for inclusion into the study), and a repeat test will be undertaken at the 3-month visit to assess subjective olfactory performance. The smell test has 3 parts—threshold (T), discrimination (D) and identification (I); this results in a composite TDI score out of 48. The test utilises a set of pens containing odours or blanks (depending on which section of the test) and using the alternative forced choice method, participants are asked to identify the presence of the odour (threshold), the odd odour out of 3 (discrimination) or the correct odour from 4 options (identification), as the pens are waved under their nose. This test takes 20–25 min to complete. The olfactory disorders questionnaire takes about 5–10 min to complete, assesses quality of life impact, and has been previously validated by the CI in the UK setting [73].

### Brain-derived neurotrophic factor (BDNF)

See the “Non-adherence and non-retention” section below.

## Participant timeline

### Visit schedule, baseline, and follow-up assessments

#### Consent or assent

Patients will be provided with a Patient Information Sheet (PIS) and given time to read it fully. Following a discussion with a medically qualified investigator or suitable trained and authorised delegate, any questions will be satisfactorily answered and if the participant is willing to participate, written informed consent will be obtained. During the consent process, it will be made completely and unambiguously clear that the participant is free to refuse to participate in all or any aspect of the trial, at any time and for any reason, without incurring any penalty or affecting their treatment.

Consent will be re-sought if new information becomes available that affects the participant’s consent in any way. This will be documented in a revision to the patient information sheet and the participant will be asked to sign an updated consent form. These will be approved by the ethics committee prior to their use.

A copy of the approved consent form is available from the NCTU trial team.

#### Patient pathway

Patients will attend their baseline visit and then have blood samples taken for BDNF. See recruitment details below; these arrangements may depend on the influence of the COVID-19 pandemic. If necessary, in order to avoid the usual endoscopic examination, potential participants may be screened by telephone and if they do not have other rhinological symptoms (such as nasal blockage, rhinorrhoea, and facial pressure), then they will proceed into the study, with the caveat that if the subsequent MRI scan shows inflammatory sinonasal disease, they will be excluded from the study at that point and replaced with another recruit in order to achieve the planned sample size. We anticipate the chances of finding sinonasal disease after screening will be 3% or less [70], and thus may only affect 1 or 2 participants.

After completion of the baseline visit (1.5–2 h), participants will commence their nasal drops as instructed by the research team. They will be invited to reattend after 3 months for a repeat assessment. After the second visit, their participation in the study will be complete. See the flowchart below for further details.

#### Early stopping of follow-up

If a participant chooses to discontinue their trial treatment, they should continue to be followed up as closely as possible to the follow-up schedule defined in the protocol, providing they are willing. They should be encouraged and facilitated not to leave the whole trial, even though they no longer take the trial treatment. If, however, the participant exercises the view that they no longer wish to be followed up, this view must be respected, and the participant must withdraw entirely from the trial. NCTU should be informed of the withdrawal in writing using the appropriate APOLLO trial documentation. Data already collected will be kept and included in analyses according to the intention-to-treat principle for all participants who stop follow-up early. Participants who stop trial follow-up early will be replaced (but their data utilised unless they request withdrawal of data).

#### Loss to follow-up

Participants who are lost to follow-up (despite attempts to contact them on 3 occasions), will be withdrawn from the study.

#### Trial closure

The end of the trial is defined as 12 months following the last follow-up visit of the last patient randomised, to allow for data entry and data cleaning activities to be completed.

### Sample size

Fifty-seven participants who are eligible and have provided informed consent will be recruited and randomised on a 2:1 ratio, with 38 allocated to receive the intervention and 19 to receive placebo. This sample size will aim to detect a difference of 20 mm^3^ in OBV with 90% power based on a 2:1 allocation ratio, and based on a standard deviation of 20 mm^3^ [69] with an assumed 10% drop-out rate accounting for the above MRI-based exclusions.

### Recruitment

Recruitment will take place over 12 months. Patients will be identified through the Smell & Taste Clinic at the James Paget University Hospital as well as from members of Fifth Sense who will be alerted via membership channels including social media and mailshots to contact the study team. Those identified from either source will be sent the relevant patient information pack (by mail/e-mail/website download). The CI and RN will screen potential participants to check their eligibility Those who contact the team via Fifth Sense will be asked to seek a GP referral to the Smell & Taste Clinic to enable recruitment. Depending on COVID-19-related restrictions at the time of commencing the study, the initial screening may be undertaken via telephone with the final eligibility checks undertaken at the baseline study visit at the UEA Brain Imaging Centre (UBIC) when they undergo their first MRI scan. In this latter scenario, the baseline Sniffin’ Sticks test will also be performed at this visit by the research associate. All necessary precautions for COVID-19 screening will be undertaken and national and local policies will be adhered to for any face-to-face contact, depending on the measures in place at the time of study commencement. Enrolment and informed consent will be undertaken by the CI and confirmed by the RA at UBIC.

### Assignment of interventions

#### Sequence generation

Participants will be randomly assigned to either the intervention or the placebo group with a 2:1 allocation as per a computer-generated randomisation schedule. The randomisation will be performed after the initial baseline MRI scan to ensure the eligibility criteria are fully met.

#### Allocation concealment mechanism and implementation

The random allocation order will be generated before the trial begins and concealed from the research team by a CTU statistician. An interactive web randomisation system (REDCap) will be used to generate a “pack ID” for the dispensing IMP for the allocation of participants to one of the two groups after the informed consent and baseline measures have been completed. The method of using pack identification numbers ensures the blind is maintained and medication can be dispensed by blinded members of the team. The patient will be allocated a participant number at the time of consent. When the results of the baseline tests, and all other pre-designated questions are completed in the CRF, the allocated staff will then have access to the randomisation process for that participant. The pack ID will be revealed and linked to that participant number.

#### Blinding (masking)

All participants, care providers, and outcome assessors will be blinded to the treatment allocation. Accidental unblinding will be logged and monitored to ensure that appropriate steps are taken to prevent this from reoccurring. The data analysts will not be blinded as they are required to know the group for the preparation of the DMEC reports.

#### Emergency unblinding

Where possible, requests for emergency unblinding of individuals should be made via NCTU in agreement with the Chief Investigator, will be sought. However, in circumstances where there is insufficient time to make this request or for agreement to be sought, the treating clinician can make the decision to unblind immediately. This can be done via the study database. The Chief Investigator will have special logins which will allow unblinding of individual patients. This is closely audited within the database management system. All instances of unblinding should be recorded and reported to NCTU by the local principal investigator, including the identity of all recipients of the unblinding information.

### Data collection methods

#### Confidentiality

Each participant will be given a unique trial Participant IDentification number (PID). Data will be collected at the time-points indicated in the Trial Schedule. The preferred method of data collection is direct online entry of data onto the central database, stored on servers based at NCTU by members of the APOLLO trial team working within each research site. Data may be entered onto paper Case Report Forms (CRFs) prior to entry into the database. Staff will receive training on data collection and use of the online system.

Data collection, data entry, and queries raised by a member of the APOLLO trial team will be conducted in line with the NCTU and trial-specific Data Management Standard Operating Procedure. Identification logs, screening logs, and enrolment logs will be kept at the trial site in a locked cabinet within a secured room.

Clinical trial team members will receive trial protocol training. All data will be handled in accordance with the Data Protection Act 2018 and the General Data Protection Regulation (GDPR) (EU) 2016/679.

Participant identifiable data will be stored on a Participants Database to enable patients to be contacted by site staff for the purpose of contacting participants and sending questionnaires; and the central trial team for the purpose of sending newsletters during the trial. There will be a clear logical separation of participant-identifiable data from the trial data.

#### Data collection

Data will be entered under the participants’ PID number onto the central database stored on the servers based at NCTU. Study data are collected and managed using REDCap [74, 75] electronic data capture tools hosted at Norwich Clinical Trials Unit (NCTU). REDCap (Research Electronic Data Capture) is a secure, web-based software platform designed to support data capture for research studies, providing audit trails for tracking data manipulation and export procedures and automated export procedures for seamless data downloads to common statistical packages.

Access to the database will be via unique, individually assigned (i.e. not generic) usernames and passwords, and only be accessible to members of the APOLLO trial team at NCTU, and external regulators if requested. The servers are protected by firewalls and are patched and maintained according to best practices. The physical location of the servers is protected physically and environmentally in accordance with the University of East Anglia’s General Information Security Policy 3 (GISP3: Physical and environmental security).

#### Data management

The database and associated code will be developed by NCTU Data Management, in conjunction with the APOLLO trial team. The database software provides a number of features to help maintain data quality, including maintaining an audit trail, allowing custom validations on all data, allowing users to raise data query requests, and search facilities to identify validation failure/missing data.

After completion of the trial, the database will be retained on the servers of NCTU for on-going analysis of secondary outcomes.

The identification, screening, and enrolment logs, linking participant identifiable data to the pseudo-anonymised PID, will be held locally by the trial site. This will either be held in written form in a locked filing cabinet or electronically in a password-protected form on hospital computers. After completion of the trial, the identification, screening, and enrolment logs will be stored securely by the sites for 5 years unless otherwise advised by NCTU.

#### Non-adherence and non-retention

The consent form will explain that if a participant wishes to withdraw from the study the data and samples acquired prior to that point will be retained. Reason for withdrawal will be recorded, if given, as will loss to follow up.

Non-adherence to trial medication will be assessed by weighing the bottles for residual volume when returned to the RA at the 3-month visit; the pre-trial bottle weight will be recorded prior to dispensing. We will also collect blood samples to measure brain-derived neurotrophic factor (BDNF) as a proxy for a measure of the use of vitamin A. Blood samples will be taken by a research nurse at the Norfolk and Norwich University Hospital (NNUH) clinical research facility at both study visits using a S-Monovette 7.5 ml Z tube (with clotting activator) and left to coagulate for 30 min at room temperature and then centrifuged at 2000 × *g* for 10 min. The serum supernatant will then be placed into an ELISA (enzyme-linked immunosorbent assay) for BDNF. These samples will be stored at − 80 °C in a Biorepository until all the samples are collected. The assays will be undertaken at the end of the trial. Once the final outcomes are reported, the samples will be destroyed.

Where patients are unable or unwilling to continue to undertake study measurements/activities, the decision as to whether the participant should continue with trial treatment and/or follow-up assessments will be at the discretion of the CI.

### Statistical methods

#### Outcomes

The comparison between groups will be based on the intention-to-treat population. The OBV will be compared between the two groups using a two-sample *t*-test. The brain imaging data will be analysed in SPM12 (UCL, London, UK) [76], based on probabilistic maps of the region of interest derived from a meta-study [77], as well as anatomical markers [78, 79]. In order to address the primary outcome measure; changes in OBV, will be compared to the control group using a two-sample test of outcome measures adjusted for baseline. The second outcome measure of changes in orbitofrontal sulcus depth will also be analysed in the same manner. To understand the wider impact of anosmia on brain function we will use resting state fMRI analysis with novel brain network and machine learning pipelines [79]. In a similar approach to Lee et al. who investigated olfactory functional networks in Parkinson’s disease dementia and Alzheimer’s dementia [80, 81], we will use a regions of interest seed-based approach for the comparison of functional changes in the resting-state networks. The seed regions of interest we aim to investigate are the olfactory bulb, olfactory tract, piriform cortex, and orbitofrontal cortex (OFC) between groups. While OBV measurement has been widely used to investigate olfactory diseases, white matter integrity analyses are more recent. DTI analyses allow the evaluation of different fibre properties in white matter. Güllmar et al. showed that improvement of olfaction in chronic rhinosinusitis patients following surgery correlated with olfactory performance and DTI measures of cortical change [82]. In our study, the diffusion MRI analysis will be conducted with a reproducible, robust, and efficient dMRI processing pipeline [83]. We will also plan to use a diffusion tensor imaging sequence for the olfactory bulb and then use a white-matter skeleton co-registration approach (e.g. tract-based spatial statistics) to reduce inter-individual differences [84]. The use of advanced machine learning algorithms will allow us to go beyond simple correlations to make predictions and rank the effects of the intervention [84–86]. The ASL data will be analysed using an automated pipeline which includes optimised pre-processing with partial volume correction, vascular artifact reduction as well as extensive quality control and data provenance [87].

#### Statistical analysis plan

A full SAP will be produced prior to the analysis of any data. The SSC will be given the opportunity to comment on the SAP prior to it being signed-off by the CI and lead statistician.

#### Additional analyses

An adjusted analysis will also be conducted adjusting for the corresponding baseline measure using a general linear model. A similar analysis to the OBV and sulcus measurement analysis will be used for the TDI score from the Sniffin’ Sticks psychophysical smell test and the ODQ. If the assumptions of the tests are not met, then non-parametric approaches will be used. We will also assess the correlation between the OBV measurements and the TDI scores using Pearson’s correlation coefficient.

#### Analysis population

The intention-to-treat population will consist of all participants who were randomised who have outcome data in the group that they were allocated to, regardless of adherence. No multiple imputation will be undertaken for this study.

#### Missing data

Patterns of missing data will be described, but no imputation will be undertaken in this early phase study.

### Data monitoring

**Fig 1:**
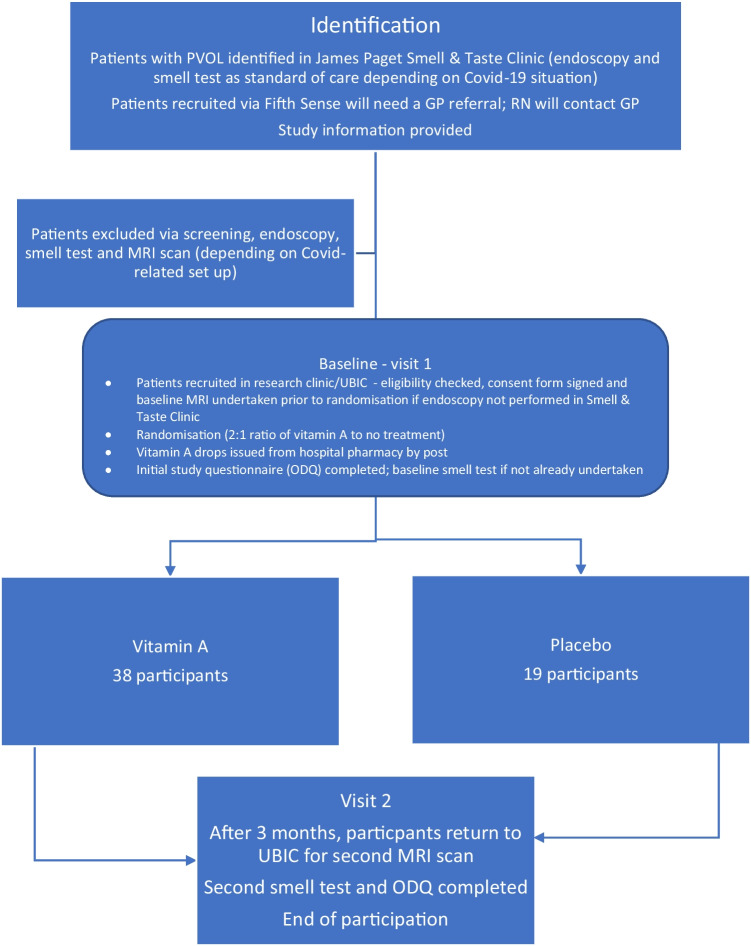
Vitamin A study flowchart

Details of the committee personnel can be found in Fig. 1 in  Appendix.

#### Data monitoring oversight

The trial will be led by the chief investigator working with the co-applicants who provide expertise in statistics, methodology, data management, functional imaging, and computational modelling. A research associate (RA) will be employed to undertake the day-to-day running of the study. Study activities and responsibilities will be documented in an overarching trial management agreement and signed by the James Paget University Hospital, CI, and the Clinical Research Network. The RA will maintain essential documentation with a study Master File and the CI will manage the regulatory and ethics approval process.

The Project Management Group (CI, RA, research nurse, other co-applicants as needed) research team will meet regularly (monthly during the setup phase then 2 monthly during the recruitment phase) to draft, review, and approve key essential documents including any documentation needed for research governance. The study steering committee will meet twice a year and include all co-applicants and affiliated research team members including our PPI representative. Online meeting platforms will be used where needed. Ad hoc meetings will be added where needed for any issues arising including safety monitoring. The Independent Steering Committee will encompass the role of a safety committee for the purposes of this trial.

#### Indications for trial closure

The trial may be stopped before completion for the following reasons:

- On the recommendation of the SSC (Study Steering Committee)
- On the recommendation of the sponsor and CI
- At request from the funder

#### Adverse event reporting and monitoring

Definitions of harm of the EU Directive 2001/20/EC Article 2 based on the principles of ICH GCP apply to this trial. We will notify the REC and sponsor of any unexpected serious adverse reactions. We will adhere to the Health Research Authority guidelines [88].

All adverse events will be recorded in the database, to monitor for harm and allow for an ongoing comparison between arms for the data monitoring and safety committee in any subsequent RCT. All non-serious AEs and ARs, whether expected or not, should be recorded in the patient’s medical notes and reported in the database. Serious Adverse Events (SAEs) and Serious Adverse Reactions (SARs) must be notified to NCTU and the sponsor within 24 h. When an AE or AR occurs, the investigator responsible for the care of the participant must first assess whether the event is serious. If the event is classified as ‘serious’ then it will be reported in an expedited manner using the study-specific reporting form. The CI (or delegate) will assess whether the event could be related to trial treatment.

If there is at least a possible involvement of the trial intervention (including placebo), the CI must assess the expectedness of the event. An unexpected adverse reaction/event is one that is not reported in the approved version of the Reference safety Information (RSI) in the Vitadral SmPC, or one that is more frequently reported or more severe than previously reported. If a SAR is assessed as being unexpected it becomes a SUSAR (suspected, unexpected, serious adverse reaction) and REC reporting guidelines apply. If the sponsor and CI deem the event relevant, it will also be reported to the MHRA via their Yellow Card scheme.

The SSC in their role as Safety Committee will be provided with all safety data, along with the corresponding treatment arm, by the trial statistician. The committee can advise that the trial is stopped early in the unlikely event that there are concerns of harm to the participants.

We will withdraw any female participant who becomes pregnant. Female participants will be asked to inform the RA if they become pregnant during the course of the study.

#### Quality assurance and control

The Quality Assurance (QA) and Quality Control (QC) considerations for the APOLLO trial are based on the standard NCTU Quality Management Policy that includes a formal Risk Assessment, and that acknowledges the risks associated with the conduct of the trial and proposals of how to mitigate them through appropriate QA and QC processes. Risks are defined in terms of their impact on the rights and safety of participants; project concept including trial design, reliability of results, and institutional risk; project management; and other considerations.

NCTU staff will review Case Report Form (CRF) data for errors and missing key data points. The trial database will also be programmed to generate reports on errors and error rates. Essential trial issues, events, and outputs, including defined key data points, will be detailed in the APOLLO trial Data Management Plan.

A Quality Management and Monitoring Plan (QMMP) will detail monitoring and IMP accountability procedures.

#### Study team

A research associate (RA) will be employed to undertake the day-to-day running of the study. A Senior Trial Manager (STM) at Norwich CTU will assist the CI and RA with study planning, study set-up prior to the appointment of the RA, oversight of study management, and adherence to CTU practices. The STM provides expertise in trial design, methodology, and conduct throughout the project. A Senior Statistician at Norwich CTU will provide advice throughout the study and statistical oversight and will be supported by a project statistician throughout the study.

#### Study management group

A study management group (SMG) will be set up to assist with developing the design, co-ordination, and strategic management of the trial. The SMG includes the members of the Study Team as well as other co-applicants who provide expertise in statistics, methodology, data management, functional imaging, computational modelling, and an independent PPI representative. The membership, frequency of meetings, activity (including trial conduct and data review), and authority will be covered in the SMG terms of reference.

The SMG will meet regularly (monthly during the setup phase then 2 monthly during the recruitment phase) to draft, review, and approve key essential documents including any documentation needed for research governance.

#### Independent study steering committee

The Independent Study Steering Committee (SSC) will include two experienced physicians independent of the trial and is the independent group responsible for oversight of the study in order to safeguard the interests of participants. The SSC provides advice to the CI, NCTU, the funder, and sponsor on all aspects of the trial. The membership, frequency of meetings, activity (including trial conduct and data review), and authority will be covered in the SSC terms of reference. Due to the nature of the trial proposed, the SSC will incorporate the role of a safety committee as defined below:

- To detect any trends, such as increases in un/expected events, and take appropriate action
- To seek additional advice or information from investigators where required
- To evaluate the risk of the trial continuing and take appropriate action where necessary

### Trial sponsor

The University of East Anglia is the trial sponsor. The role of the sponsor is to take on responsibility for securing the arrangements to initiate, manage, and finance the trial. The Sponsor is responsible for ensuring that the study meets the relevant standards and makes sure that arrangements are put and kept in place for management, monitoring, and reporting. The University of East Anglia has delegated some Sponsor’s activities to the CI and NCTU, these are documented in the collaboration agreement and delegation of responsibilities.

## Ethics and dissemination

### Research ethics approval

Before initiation of the trial at any clinical site, the protocol, all informed consent forms and any material to be given to the prospective participant will be submitted to the relevant ethics committee for approval. Any subsequent amendments to these documents will be submitted for further approval.

The rights of the participant to refuse to participate in the trial without giving a reason must be respected. After the participant has entered the trial, the clinician remains free to give alternative treatment to that specified in the protocol, at any stage, if s/he feels it to be in the best interest of the participant. The reasons for doing so must be recorded. After randomisation, the participant must remain within the trial for the purpose of follow-up and data analysis according to the treatment option to which they have been allocated. However, the participant remains free to change their mind at any time about the protocol treatment and follow-up without giving a reason and without prejudicing their further treatment. The study has received approval from the West Midlands–Black Country Research Ethics Committee, reference number 21/WM/0179. The study has also received Health Regulation Authority approval and all sites will be required to provide confirmation of capacity and capability before recruitment starts.

### Competent authority approvals

The APOLLO trial is not a Clinical Trial of an Investigational Medicinal Product (IMP) as defined by the EU Directive 2001/20/EC. Therefore, a CTA is not required in the UK.

### Confidentiality

Any paper copies of personal trial data will be kept at the participating site in a secure location with restricted access. Confidentiality of patient’s personal data is ensured by not collecting patient names on CRFs and limiting access to personal information held on the database at NCTU. At trial enrolment, the patient will be issued a participant identification number and this will be the primary identifier for the patient, with secondary identifiers of month and year of birth and initials.

The patient’s consent form will carry their name and signature. These will be kept at the trial site, a copy may be sent to NCTU for monitoring purposes. This copy will be destroyed once checks are complete. Consent forms will not be kept with any additional patient data.

### Declaration of interests

The investigators named on the protocol have no financial or other competing interests that impact their responsibilities towards the scientific value or potential publishing activities associated with the trial.

There are no financial or other competing interests for the research team.

### Access to data

Requests for access to trial data will be considered, and approved in writing where appropriate, after formal application to the SMG. Considerations for approving access are documented in the SMG Terms of Reference. The final trial dataset will be held at Norwich CTU on a secure server and will be accessible by the research team. UEA will own the foreground IP (as employer of the Chief Investigator) and JPUH will have rights via a royalty-free licence to use for their own non-commercial purposes and patient benefit. Any exploitation shall be managed through the University’s Intellectual Property Team who shall put into place appropriate royalty share agreements although it is not anticipated that this will be the case as further research will be required.

### Ancillary and post-trial care

There are no plans to offer trial treatment to individuals participating in this study after its conclusion. Vitamin A supplementation is available without a prescription should the participant wish to continue with this following the study. Any participants needing additional care will be returned to the Norfolk Smell & Taste Clinic for management. This will also apply to those who have persistent olfactory dysfunction at the end of the study.

### Dissemination policy

As part of the wider body of work of the APOLLO programme, we anticipate dissemination through traditional channels including conference presentations, abstracts, and open-access peer-reviewed publications. These will be targeted at both specialist and generalist groups, facilitated by roles held by members of our collaboration in relevant professional societies and guideline-producing bodies. However, beyond informal and specialist publications, we also plan to use wider communication channels to allow larger-scale dissemination to the public. When our PPI panel has facilitated a patient-friendly and comprehensible summary of study findings, these will be provided to all study participants by mailings and to the wider public via a study website. We will maximize non-professional dissemination through several multimedia outlets, including targeted mailshots, press releases to medical and general journalists, health information websites and communications media, evidence-based interest groups, conference presentations, social media, and patient interest groups/medical charities such as Fifth Sense. We anticipate the results will lead to the production of new guidelines which will be made available through websites such as ENT UK and the Royal College of Surgeons.

## Discussion

The APOLLO trial is funded by the National Institute of Health Research, Research for Patient Benefit Programme funding stream (NIHR, RfPB). The study is run by a Study Management Group (SMG) and overseen by the Independent Study Steering Committee (SSC) which includes two experienced physicians independent of the trial to safeguard the interests of participants. As listed below in Fig. 1 in  Appendix, the trial is managed by the SMG and has independent oversight from the SCC. Day-to-day trial administration is the responsibility of the Study Manager and the Chief Investigator.

## Abbreviations

AE: Adverse event
AR: Adverse reaction
ASL: Arterial spin labelling
BDNF: Brain-derived neurotrophic factor
BOLD: Blood oxygen level-dependent
CI: Chief investigator
CRF: Case Report Form
CRS: Chronic rhinosinusitis
DMC: Data Management Committee
DTI: Diffusion tensor imaging
EU: European Union
FDA: (US) Food and Drug Administration
FWA: Federal Wide Assurance
GCP: Good Clinical Practice
HRA: Health Research Authority
ICH: International Conference on Harmonisation
IMP: Investigational Medicinal Product
ITT: Intention to treat
JPUH: James Paget University Hospital NHS Foundation Trust
MHRA: Medicines and Healthcare products Regulatory Agency
(d)MRI: (Diffusion) Magnetic resonance imaging
(f)MRI: (Functional) Magnetic resonance imaging
NAEL: Notifiable adverse event
NCTU: Norwich clinical trials unit
NNUH: Norfolk and Norwich University Hospitals NHS Foundation Trust
OBV: Olfactory bulb volume
ODQ: Olfactory Disorders Questionnaire
OFC: Orbitofrontal cortex
ORN: Olfactory receptor neuron
PI: Principal investigator
PID: Participant identification number
PIS: Participant Information Sheet
PROMS: Patient-reported outcome measures
PVOL: Post-viral olfactory loss
QA: Quality assurance
QC: Quality control
QMMP: Quality Management and Monitoring Plan
RA: Research associate
RAc: Retinoic acid
R&D: Research and development
REC: Research Ethics Committee
RN: Research nurse
SAE: Serious adverse event
SAP: Statistical analysis plan
SAR: Serious adverse reaction
SPC: Summary of product characteristics
SSA: Site-specific approval
SSC: Study Steering Committee
SUSAR: Suspected unexpected serious adverse reaction
TDI: Threshold, Discrimination, Identification
TMF: Trial master file
TMG: Trial management group
TMT: Trial management team
ToR: Terms of reference
TSC: Trial steering committee
TNSTC: The Norfolk Smell & Taste Clinic
UADE: Unexpected adverse device effect
UBIC: UEA Brain Imaging Centre
UEA: University of East Anglia
